# Testing for direct genetic effects using a screening step in family-based association studies

**DOI:** 10.3389/fgene.2013.00243

**Published:** 2013-11-21

**Authors:** Sharon M. Lutz, Stijn Vansteelandt, Christoph Lange

**Affiliations:** ^1^Department of Biostatistics, University of ColoradoAurora, CO, USA; ^2^Department of Biostatistics, Harvard School of Public HealthBoston, MA, USA; ^3^Department of Applied Mathematics, Computer Science and Statistics, Ghent UniversityGhent, Belgium

**Keywords:** family-based association analysis, causal inference, genetic pathway, mediation, pleiotropy

## Abstract

In genome wide association studies (GWAS), family-based studies tend to have less power to detect genetic associations than population-based studies, such as case-control studies. This can be an issue when testing if genes in a family-based GWAS have a direct effect on the phenotype of interest over and above their possible indirect effect through a secondary phenotype. When multiple SNPs are tested for a direct effect in the family-based study, a screening step can be used to minimize the burden of multiple comparisons in the causal analysis. We propose a 2-stage screening step that can be incorporated into the family-based association test (FBAT) approach similar to the conditional mean model approach in the Van Steen-algorithm (Van Steen et al., 2005). Simulations demonstrate that the type 1 error is preserved and this method is advantageous when multiple markers are tested. This method is illustrated by an application to the Framingham Heart Study.

## Introduction

Some of the recently published genome-wide association studies identified the same genetic locus as a disease susceptibility locus for different complex diseases (Amos et al., 2008; Thorgeirsson et al., 2008). One possible mechanism is that the marker locus is pleiotropic and has genetic effects on several, different phenotypes. Determining whether the marker acts directly on each of these phenotypes or only indirectly via one or more intermediate phenotypes is an important step in understanding the biological significance of the genetic associations. In order to understand and characterize the underlying genetic effect, methods have been proposed to disentangle these potential direct and indirect genetic effects (Vansteelandt et al., 2009; Vansteelandt, 2010; Berzuini et al., 2012; Vansteelandt and Lange, 2012; VanderWeele et al., 2012). All currently available methods focus on the direct and indirect genetic effects relative to one (group of) secondary phenotypes. Because the magnitude of the indirect effect depends on how strongly these secondary phenotypes affect the primary phenotype, these methods consider adjustment for confounding of the relationship between these phenotypes by measured extraneous factors. Some of these methods quantify both the direct and indirect genetic effects, but assume that none of these extraneous confounding factors is influenced by the considered marker (VanderWeele et al., 2012). Some of these methods allow for some of the extraneous confounding factors to be influenced by the considered marker, but they merely quantify direct genetic effects (Vansteelandt et al., 2009; Vansteelandt, 2010; Berzuini et al., 2012).

Regardless of the considered framework, all available methods only test one gene at a time and need to be corrected for multiple comparisons. This concern over multiple comparisons becomes an issue in family-based genome wide association studies (GWAS). When there is a region with a strong association with both the endo-phenotype and phenotype, identifying SNPs in the region that are still associated with the phenotype of interest after accounting for the association with the endo-phenotype requires testing for a direct causal effect for every SNP in the region. In order to increase power to detect this direct genetic effect, we propose a 2-stage testing strategy to minimize the burden of multiple comparisons in the causal analysis (Van Steen et al., 2005; Murphy et al., 2008; Won et al., 2009). The application of a screening step when testing for direct genetic effects is an important advantage in this scenario where the multiple-comparison problem is a major hurdle. The power of our approach is assessed by simulation studies. We show that the type-1 error is preserved and the method is shown to be advantageous when multiple SNPs are tested for a direct effect on the phenotype of interest.

## Methods

Suppose that in the family-based study, *n* trios (offspring and both parents) have been genotyped at a specific marker locus. Assuming there is no bias due to ascertainment conditions, the variable *X*_*i*_ denotes the coded genotype of the offspring and *S*_*i*_ denotes the parental genotypes for individual *i*. If genotypic data is unavailable for the parents but genotypic information is available on the subject's siblings, the variable *S*_*i*_ denotes the sufficient statistic by Rabinowitz and Laird (2000) For offspring *i*, *Y*_*i*_ denotes the target phenotype in the association study and *K*_*i*_ denotes the secondary phenotype in the study.

**Figure 1:**
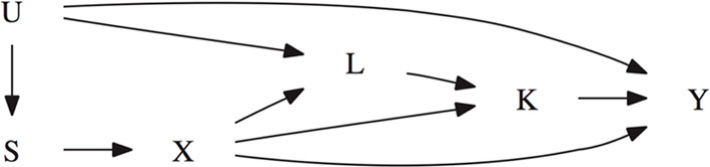
**Causal diagram illustrating the confounding of the target phenotype *Y* and the marker locus *X*.** *S* denotes the parental genotype or Rabinowitz and Laird's sufficient statistic. *K* denotes the secondary phenotype of interest. *L* allows for confounding between *K* and *Y*. *U* represents a collection of unmeasured factors that allow for confounding due to population stratification or confounding between the two phenotypes *K* and *Y*. Note that causal diagrams assume that all variables that jointly affect any two variables are included. The absence of an arrow between any two variable denotes that there is no direct causal effect. For instance, *U* has no direct causal effect on *X*.

Suppose that an association has been observed between the secondary phenotype of interest, *K*_*i*_, and the marker locus. Given this association, the goal is to test for an association between the target phenotype *Y*_*i*_ and the marker locus that cannot be explained by a possible indirect effect mediated by *K*_*i*_. To achieve this goal, data is needed on all risk factors of the secondary phenotype *K*_*i*_ that are also associated with the primary phenotype (Cole and Hernan, 2002). Let *L*_*i*_ denote this collection of measured confounding variables. Because *L* may be high-dimensional, we do not assume that it is only related with *Y* by means of a causal effect, but allow for their association to be itself confounded by potentially unmeasured factors *U*. This is shown in the causal diagram of Figure 1, where the presence of *U* additionally captures the potential for confounding of the genetic association as a result of population admixture (Vansteelandt and Lange, 2012). Throughout, in contrast to other mediation analysis techniques (namely those based on so-called natural direct and indirect effects), we will allow for the possibility that some of these confounding variables are themselves affected by the studied marker, as illustrated via the edge from *X* to *L* in the causal diagram (VanderWeele et al., 2012).

Consider model

(1) $$E\text{​}\left[Y_{i}|X_{i},K_{i},L_{i}\right]=\gamma_{0}+\gamma_{1}K_{i}+\gamma_{2}X_{i}+\gamma_{3}L_{i}$$

where γ_*j*_ for *j* = 0, 1, …, 3 denote the mean parameters and can be estimated by ordinary least squares. Note that γ_1_ represents the true effect of *K*_*i*_ on *Y*_*i*_ and not a spurious association because, by assumption, the above model includes all relevant risk factors of *K*_*i*_. In order to construct an adjustment principle that tests for a direct genetic effect of the marker locus *X* on the target phenotype *Y*, the effect of the secondary phenotype *K* has to be estimated. Vansteelandt et al. use an estimate for γ_1_ based on model (1) to adjust the phenotype *Y*_*i*_ to *Y*_*i*_ − γ_1_*K*_*i*_. A family-based association test (FBAT) on this adjusted phenotype is then a test for the direct genetic effect in the family-based setting (provided that the distribution of the test statistic acknowledges the uncertainty in the estimated effect γ_1_) (Vansteelandt et al., 2009).

To reduce the number of multiple comparisons, we adapt the conditional mean model approach in the VanSteen-algorithm (Van Steen et al., 2005) to model (1). By replacing the observed marker score in model (1) by the expected marker score conditional upon the parental genotypes or sufficient statistic, the genetic effects of locus *X*_*i*_ can be assessed without having to adjust the α-level of any subsequently computed FBATs (Lange et al., 2003a,b; Van Steen et al., 2005). Similar to the idea of the conditional mean model approach, model (1) can be rewritten by substituting *X*_*i*_ with its expected value *E*(*X*_*i*_|*S*_*i*_),

(2) $$E\text{​}\left[Y_{i}|K_{i},L_{i},S_{i}\right]=\beta_{0}+\gamma_{1}K_{i}+\beta_{2}L_{i}+\beta_{3}E\text{​}\left(X_{i}|S_{i}\right),$$

As shown in the proof given in the appendix, the parameter γ_1_ is the same in both model (1) and model (2) when the null hypothesis holds that there is no direct effect and, moreover, there is no confounding due to population substructure. For testing the null hypothesis of no direct genetic effect, model (2) can thus be used to estimate the parameter γ_1_ in a screening step without biasing the significance level since *X*_*i*_ is not included in this model, provided there is no confounding due to population substructure.

For the screening step, each subject contributes

(3) $$T_{i}^{*}=\left\{E\text{​}\left(X_{i}|S_{i}\right)\right\}\tilde{Y_{i}^{*}}$$

where $\tilde{Y_{i}^{*}}=Y_{i}-\bar{y}-\hat{\gamma_{1}^{*}}(K_{i}-\bar{k})$ and $\hat{\gamma_{1}^{*}}$ is the ordinary least squares estimate for γ_1_ in model (2), which does not involve the genetic marker *X*. $\tilde{Y_{i}^{*}}$ is not adjusted for the covariates *L*_*i*_ since including factors such as *L*_*i*_ in the phenotypic adjustment would introduce bias if the common risk factor *L*_*i*_ is associated with the DSL *X*_*i*_ (Vansteelandt et al., 2009). The parameters *y* and *k* are the observed phenotypic means of *Y* and *K* in the sample, respectively. Then the test statistic for the screening step is

(4) $$\frac{{\left(\sum_{i=1}^{n}T_{i}^{*}\right)}^{2}}{\sum_{i=1}^{n}\text{var}\tilde{\left(T_{i}^{*}\right)}}$$

where

(5) $$\tilde{T_{i}^{*}}=T_{i}^{*}-E\text{​}\left[\left\{E\text{​}\left(X_{i}|S_{i}\right)\right\}(K_{i}-\bar{k})\right]\frac{\left(K_{i}-\mu_{k}^{*(i)}\right)}{\sigma_{k}^{*2}}\epsilon_{i}^{*}$$

where $\text{var}(\tilde{T_{i}^{*})}$ is calculated based on the sample variance of $\tilde{T^{*}}$ and ϵ^*^_i_ denotes the residual from model (2). μ^*(*i*)^_*k*_ = *E*(*K*|*L*_*i*_, *E*(*X*_*i*_|*S*_*i*_)) is the predicted value for *K* under a linear regression model for *K* with the covariates *L*_*i*_ and *E*(*X*_*i*_|*S*_*i*_), and σ^*2^_*k*_ denotes the residual variance in that model. The variance correction given in Equation (5) is needed to account for estimating γ_1_ in the proposed phenotype adjustment $\tilde{Y_{i}^{*}}$ (Vansteelandt et al., 2009).

For step 1, the test statistic given in Equation (4) can be used for the screening step to pick the SNPs with the highest power since *X* is not used in this test statistic. For step 2, this smaller subset of SNPs are used to test the null hypothesis of no direct effect using the test statistic based on Equation (1) proposed by Vansteelandt et al. (2009)

(6) $$T_{i}=\{X_{i}-E(X_{i}|S_{i})\}\tilde{Y_{i}}$$

where $\tilde{Y_{i}}=Y_{i}-\bar{y}-\hat{\gamma_{1}}(K_{i}-\bar{k})$ and $\hat{\gamma_{1}}$ is the ordinary least square estimate for γ_1_ in model (1), which does involve the genetic marker X. Using this association test with the adjusted phenotype $\tilde{Y_{i}}$ as the target phenotype provides a robust and valid test for the null hypothesis that there is no direct effect between the target phenotype *Y*_*i*_ and the DSL; i.e., the association between the target phenotype *Y*_*i*_ and the DSL is solely a result of the association between the secondary phenotype *K*_*i*_ and the DSL. Adjusting for estimating γ_1_ based on model (1), the test statistic is distributed chi-square with one degree of freedom under the null hypothesis of no direct effect of *X* on *Y* and has the following form

(7) $$\frac{{\left(\sum_{i=1}^{n}T_{i}\right)}^{2}}{\sum_{i=1}^{n}\text{var}({\tilde{T}}_{i})}$$

where

(8) $${\tilde{T}}_{i}=T_{i}-E\text{​}\left[\left\{X_{i}-E(X_{i}|S_{i})\right\}K_{i}\right]\frac{\left(K_{i}-\mu_{k}^{\left(i\right)}\right)}{\sigma_{k}^{2}}\epsilon_{i}$$

where $\text{var}(\tilde{T_{i})}$ is calculated based on the sample variance of $\tilde{T}$ and ϵ_*i*_ denotes the residual from model (1). μ^(*i*)^_*k*_ = *E*(*K*|*L*_*i*_, *X*_*i*_, *E*(*X*_*i*_|*S*_*i*_)) is the predicted value for *K* under a linear regression model for *K* with the covariates *L*_*i*_, *X*_*i*_, and *E*(*X*_*i*_|*S*_*i*_), and σ^2^_*k*_ denotes the residual variance in that model. The variance correction given in Equation (8) is needed to account for estimating γ_1_ in the proposed phenotype adjustment $\tilde{Y_{i}}$ (Vansteelandt et al., 2009). Note that Equation (3) is similar to Equation (6), but Equation (6) contains the genetic marker *X*_*i*_. Similarly, Equation (5) is similar to Equation (8), but Equation (8) contains the genetic marker *X*_*i*_.

Note that under the alternative hypothesis, the association between *K* and *Y* is different in models (1) and (2), even in the absence of population admixture. Model (1) represents the causal effect of *K* on *Y* under the alternative hypothesis, but model (2) does not represent the causal effect of *K* on *Y* because there is a remaining spurious association between *X* and *Y* along the path *K* ← X → Y in Figure 1. Under the null hypothesis, this path does not exist. As a result, the proposed approach is valid for testing in the absence of population stratification, but may have less power when either the *X* → *K* or the *X* → *Y* link is strong. This scenario is explored further in the simulation section of this paper.

Because the test statistic for the screening step given in Equation (4) is susceptible to population stratification, we examined this scenario in the simulation section as well. Principal component analysis (PCA) can be used in the screening step to correct for population stratification.

## Simulations

Using simulation studies, we asses the type-1 error rate, the power, and robustness of this new approach which uses a trait that estimates γ_1_ based on model (2) in the screening step and compare it to the approach proposed by Vansteelandt et al. (2009) which uses a trait that estimates γ_1_ based on model (1). Similar to Vansteelandt et al. (2009), both methods are evaluated under various conditions. All simulations use a sample size of 1000 trios and are based on 5000 replications. The simulations are run for allele frequencies 5, 10, 15, 20, 25, 30, 35, 40, and 45%.

**Figure 2:**
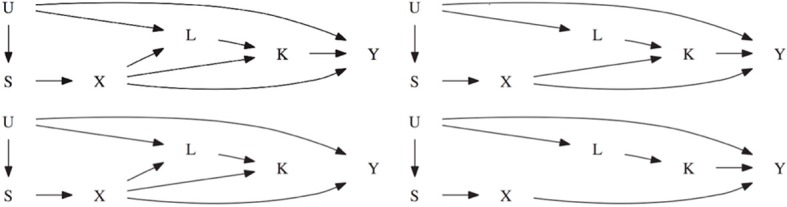
**The top left figure represents scenario 1.** The top right figure represents scenario 2 which is the same as scenario 1 except that *X* does not cause *L*. The bottom left figure represents scenario 3 which is the same as scenario 1 except that *K* does not cause *Y*. The bottom right figure represents scenario 4 which is the same as scenario 2 except that *X* does not cause *K*.

To reflect a realistic setting, the data is simulated to reflect covariances found in the Framingham Heart Study (Herbert et al., 2006). The phenotype of interest *Y* is simulated such that it resembles FEV1. The secondary phenotype *K* resembles weight and the set of common confounding variables resemble height and age. As seen in Figure 2, the first scenario assumes there is a direct genetic effect of the marker on the intermediate phenotype *K* and on the common covariate *L*. Each genetic effect has a locus specific heritability of 1%. The intermediate phenotype *K* explains 1% of the phenotypic variation in *Y*, creating an association between the SNP and *Y*. The second scenario is similar to the first scenario except that there is no genetic effect on the confounder *L*. The genetic association with the intermediate phenotype *K* is still present. The third scenario is similar to the first scenario except there is no association between *K* and *Y*. The fourth scenario is similar to the second scenario except that there is no genetic effect on the intermediate phenotype *K*.

As seen in Table 1, the type-1 error rate is similar whether model (1) or model (2) is used to estimate γ_1_. For lower allele frequencies, under scenario 1 and 3, the type-1 error rate is 1–2% higher than expected. For higher allele frequencies under all four scenarios, the type-1 error rate is 0.5% lower than expected. In general, the type-1 error rate is close to 0.05 regardless of how γ_1_ is estimated. As seen in Table 2, the power is similar whether model (1) or model (2) is used to estimate γ_1_ assuming no population admixture. For lower allele frequencies, the method by Vansteelandt et al. (2009) has higher power and for higher allele frequencies the proposed method has higher power. However, this difference in power is negligible; the power never differs by more than 2%.

**Table 1:** **This table displays the type-1 error rate for the test statistics using Model 1 [the Vansteelandt et al. test statistic (Vansteelandt et al., 2009)] or Model 2 (the screening test statistic) to estimate γ_1_ for different allele frequencies**.

**Allele frequency (%)**	**Type-1 error rate when 1 SNP is tested**								
	**5**	**10**	**15**	**20**	**25**	**30**	**35**	**40**	**45**
Scenario 1: Model 1	0.071	0.059	0.049	0.047	0.045	0.047	0.049	0.051	0.050
Scenario 1: Model 2	0.069	0.058	0.048	0.046	0.046	0.046	0.049	0.050	0.051
Scenario 2: Model 1	0.044	0.045	0.045	0.045	0.045	0.045	0.045	0.043	0.045
Scenario 2: Model 2	0.045	0.044	0.045	0.045	0.045	0.043	0.045	0.043	0.045
Scenario 3: Model 1	0.058	0.048	0.043	0.045	0.045	0.046	0.044	0.047	0.044
Scenario 3: Model 2	0.052	0.050	0.044	0.046	0.044	0.046	0.045	0.047	0.046
Scenario 4: Model 1	0.044	0.045	0.045	0.043	0.046	0.044	0.045	0.045	0.042
Scenario 4: Model 2	0.044	0.044	0.045	0.043	0.046	0.044	0.046	0.045	0.042

**Table 2:** **This table displays the power for the test statistics using Model 1 [the Vansteelandt et al. test statistic (Vansteelandt et al., 2009)] or Model 2 (the screening test statistic) to estimate γ_1_ for different allele frequencies**.

**Allele frequency (%)**	**Power when 1 SNP is tested**								
	**5**	**10**	**15**	**20**	**25**	**30**	**35**	**40**	**45**
Scenario 1: Model 1	0.264	0.363	0.448	0.504	0.576	0.629	0.669	0.692	0.706
Scenario 1: Model 2	0.241	0.361	0.444	0.508	0.581	0.633	0.671	0.696	0.710
Scenario 2: Model 1	0.180	0.302	0.406	0.492	0.564	0.610	0.649	0.667	0.686
Scenario 2: Model 2	0.180	0.302	0.408	0.491	0.563	0.610	0.646	0.666	0.685
Scenario 3: Model 1	0.265	0.365	0.449	0.504	0.581	0.632	0.669	0.696	0.712
Scenario 3: Model 2	0.246	0.361	0.451	0.510	0.586	0.634	0.671	0.699	0.716
Scenario 4: Model 1	0.175	0.304	0.408	0.499	0.558	0.607	0.647	0.671	0.681
Scenario 4: Model 2	0.174	0.303	0.407	0.498	0.557	0.605	0.648	0.672	0.682

The advantage of our approach becomes clear when testing multiple SNPs. Table 4 shows how the power to detect the causal SNP for our approach compares to Vansteelandt et al. (2009) when one SNP has a direct effect on the phenotype as simulated above in Table 2 and 49 other SNPs are not associated with the phenotype of interest. Table 1 shows the type-1 error rate in this scenario where the one SNP has an indirect effect on the phenotype as simulated above in Table 1 and 49 other SNPs are not associated with the phenotype of interest or any of the other phenotypes. Table 6 shows how the power to detect the causal SNP for our approach compares to Vansteelandt et al. (2009) when one SNP has a direct effect on the phenotype as simulated above in Table 2 and 99 other SNPs are not associated with the phenotype of interest. Table 5 shows the type-1 error rate in this scenario where the one SNP has an indirect effect on the phenotype as simulated above in Table 1 and 99 other SNPs are not associated with the phenotype of interest or any of the other phenotypes.

Our approach allows for a screening step similar to the Van Steen algorithm (Van Steen et al., 2005) where the top 3 SNPs out of 50 and the top 5 SNPs out of 100 with the highest test statistic given by Equation (4) are chosen. We chose 3 SNPs out of 50 and 5 SNPs out of 100 since this is roughly 5% of the SNPs. After the top 3 or 5 SNPs are chosen based on the screening step, the test statistic described in Equation (7) is used to obtain a *p*-value which is compared to α/3 and α/5, respectively. We compare our approach with the screening step to the approach by Vansteelandt et al. (2009) with a Sidak correction. Since our approach allows for a screening step, we are better able to detect the SNP that has a direct causal effect on the target phenotype as seen in Tables 4, 6.

Note that the power in Tables 4, 6 is lower than that in Table 2 which is expected since multiple SNPs are tested. For more common allele frequencies, the power of using the proposed method with a screening step is more than double that of the Vansteelandt algorithm with a Sidak correction while the type-1 error rates are similar as seen in Tables 3, 5. Therefore, if multiple SNPs are tested, the proposed approach has better power to detect the SNP that has a direct effect on the phenotype of interest.

**Table 3:** **This table displays the significance rate when one SNP does not have a direct effect on the phenotype Y but acts as seen in Figure 2 without the arrow from X to Y and 49 SNPs are not associated with the phenotype Y**.

**Allele frequency (%)**	**Type-1 error rate when 50 SNPs are tested**								
	**5**	**10**	**15**	**20**	**25**	**30**	**35**	**40**	**45**
Scenario 1: Model 1	0.0018	0.0008	0.0008	0.0006	0.0004	0.0006	0.0008	0.0006	0.0010
Scenario 1: Model 2	0.0014	0.0006	0.0002	0.0006	0.0012	0.0012	0.0006	0.0012	0.0006
Scenario 2: Model 1	0.0014	0.0006	0.0008	0.0012	0.0004	0.0008	0.0004	0.0008	0.0002
Scenario 2: Model 2	0.0004	0.0010	0.0012	0.0016	0.0012	0.0006	0.0010	0.0004	0.0006
Scenario 3: Model 1	0.0018	0.0006	0.0008	0.0014	0.0006	0.0010	0.0008	0.0008	0.0002
Scenario 3: Model 2	0.0014	0.0006	0.0008	0.0016	0.0012	0.0010	0.0012	0.0004	0.0006
Scenario 4: Model 1	0.0014	0.0006	0.0008	0.0012	0.0004	0.0008	0.0004	0.0008	0.0002
Scenario 4: Model 2	0.0008	0.0010	0.0013	0.0016	0.0012	0.0006	0.0010	0.0004	0.0006

*Model 1 is used to estimate γ_1_ with a Sidak correction and Model 2 is used estimate γ_1_ with a screening step where the three SNPs with the largest test statistic given by Equation (8) are tested*.

Since the proposed approach is valid for testing, but may have less power when either the *X* → *K* or the *X* → *Y* link is strong, we looked at the effect of increasing the association between *X* and *K* when *K* influences *Y* (*X* → *K*) and *X* and *Y* (*X* → *Y*). We increased the correlation between *X* and *K* from 0.025 to 0.05 and then 0.075. We also increased the correlation between *X* and *Y* from 0.05 to 0.10 and then 0.15. The power of both statistics remained very close. At most, the power of the Vansteelandt et al. statistic (Vansteelandt et al., 2009) was 0.9% better than our approach.

Since the test statistic for the screening step given in Equation (4) is susceptible to population stratification, we examined a few scenarios where population stratification was present. We simulated half of the subjects to have allele frequency of 5, 5, 20, and 40% and the other half of the subjects to have allele frequency of 10, 45, 25, and 45%, respectively. Similar to Tables 3, 4, one SNP has a direct effect on the phenotype of interest and 49 other SNPs are not associated with the phenotype of interest in Tables 7, 8. Similar to Tables 5, 6, one SNP has a direct effect on the phenotype of interest and 99 other SNPs are not associated with the phenotype of interest in Tables 9, 10. As seen in Tables 7, 9, the type-1 error rates are similar for both methods. As seen in Tables 8, 10, even though there is some population stratification present, the proposed method with a screening step still performs better than the Vansteelandt algorithm, especially when the allele frequencies are more common.

**Table 4:** **This table displays the power when one SNP has a direct effect on the phenotype Y and 49 SNPs are not associated with the phenotype Y**.

**Allele frequency (%)**	**Power when 50 SNPs are tested**								
	**5**	**10**	**15**	**20**	**25**	**30**	**35**	**40**	**45**
Scenario 1: Model 1	0.031	0.039	0.073	0.075	0.120	0.150	0.176	0.191	0.191
Scenario 1: Model 2	0.038	0.073	0.133	0.188	0.255	0.321	0.356	0.368	0.431
Scenario 2: Model 1	0.013	0.030	0.040	0.074	0.110	0.112	0.158	0.162	0.172
Scenario 2: Model 2	0.015	0.056	0.117	0.18	0.236	0.292	0.344	0.356	0.378
Scenario 3: Model 1	0.031	0.039	0.074	0.083	0.121	0.130	0.185	0.191	0.201
Scenario 3: Model 2	0.038	0.073	0.136	0.194	0.257	0.312	0.368	0.370	0.445
Scenario 4: Model 1	0.012	0.030	0.063	0.076	0.110	0.113	0.159	0.176	0.177
Scenario 4: Model 2	0.015	0.057	0.107	0.181	0.235	0.290	0.344	0.376	0.416

*Model 1 is used to estimate γ_1_ with a Sidak correction and Model 2 is used estimate γ_1_ with a screening step where the three SNPs with the largest test statistic given by Equation (8) are tested*.

**Table 5:** **This table displays the significance rate when one SNP does not have a direct effect on the phenotype Y but acts as seen in Figure 2 without the arrow from X to Y and 99 SNPs are not associated with the phenotype Y**.

**Allele frequency (%)**	**Type-1 error rate when 100 SNPs are tested**								
	**5**	**10**	**15**	**20**	**25**	**30**	**35**	**40**	**45**
Scenario 1: Model 1	0.0010	0.0006	0.0004	0.0006	0.0007	0.0006	0.0002	0.0004	0.0005
Scenario 1: Model 2	0.0008	0.0004	0.0004	0.0006	0.0006	0.0006	0.0008	0.0002	0.0006
Scenario 2: Model 1	0.0006	0.0000	0.0008	0.0000	0.0000	0.0004	0.0002	0.0006	0.0002
Scenario 2: Model 2	0.0004	0.0004	0.0008	0.0002	0.0004	0.0006	0.0010	0.0004	0.0008
Scenario 3: Model 1	0.0010	0.0010	0.0002	0.0004	0.0000	0.0004	0.0002	0.0008	0.0000
Scenario 3: Model 2	0.0008	0.0004	0.0002	0.0002	0.0002	0.0006	0.0002	0.0002	0.0004
Scenario 4: Model 1	0.0006	0.0003	0.0004	0.0006	0.0007	0.0006	0.0002	0.0004	0.0005
Scenario 4: Model 2	0.0002	0.0004	0.0004	0.0006	0.0006	0.0006	0.0008	0.0002	0.0006

*Model 1 is used to estimate γ_1_ with a Sidak correction and Model 2 is used estimate γ_1_ with a screening step where the five SNPs with the largest test statistic given by Equation (8) are tested*.

**Table 6:** **This table displays the power when one SNP has a direct effect on the phenotype Y and 99 SNPs are not associated with the phenotype Y**.

**Allele frequency (%)**	**Power when 100 SNPs are tested**								
	**5**	**10**	**15**	**20**	**25**	**30**	**35**	**40**	**45**
Scenario 1: Model 1	0.014	0.028	0.049	0.048	0.084	0.109	0.111	0.147	0.142
Scenario 1: Model 2	0.021	0.056	0.099	0.136	0.196	0.262	0.277	0.332	0.351
Scenario 2: Model 1	0.004	0.018	0.040	0.055	0.076	0.099	0.098	0.116	0.123
Scenario 2: Model 2	0.014	0.042	0.088	0.145	0.178	0.246	0.249	0.284	0.332
Scenario 3: Model 1	0.018	0.028	0.038	0.049	0.087	0.094	0.112	0.128	0.139
Scenario 3: Model 2	0.023	0.057	0.099	0.137	0.198	0.229	0.283	0.315	0.368
Scenario 4: Model 1	0.006	0.018	0.040	0.041	0.076	0.086	0.098	0.116	0.123
Scenario 4: Model 2	0.011	0.042	0.088	0.126	0.178	0.209	0.249	0.284	0.332

*Model 1 is used to estimate γ_1_ with a Sidak correction and Model 2 is used estimate γ_1_ with a screening step where the five SNPs with the largest test statistic given by Equation (8) are tested*.

**Table 7:** **This table displays the significance level when one SNP has an indirect effect on the phenotype Y as seen in Figure 2 without the arrow from X to Y and 49 SNPs are not associated with the phenotype Y**.

**Allele frequency**	**Type-1 error rate when the following population stratification is present**			
	**5 and 10%**	**5 and 45%**	**20 and 25%**	**40 and 45%**
Scenario 1: Model 1	0.0012	0.0014	0.0011	0.0013
Scenario 1: Model 2	0.0006	0.0006	0.0004	0.0005
Scenario 2: Model 1	0.0010	0.0006	0.0004	0.0006
Scenario 2: Model 2	0.0012	0.0013	0.0018	0.0020
Scenario 3: Model 1	0.0009	0.0002	0.0004	0.0011
Scenario 3: Model 2	0.0008	0.0012	0.0016	0.0008
Scenario 4: Model 1	0.0006	0.0014	0.0008	0.0009
Scenario 4: Model 2	0.0009	0.0006	0.0006	0.0012

*Model 1 is used to estimate γ_1_ with a Sidak correction and Model 2 to is used estimate γ_1_ in a screening step where the three SNPs with the largest test statistic given by Equation (4) are tested. Population stratification is present such that half of the subjects have one of the allele frequencies listed and the other half of the subjects have the other allele frequency listed*.

**Table 8:** **This table displays the power when one SNP has a direct effect on the phenotype Y and 49 SNPs are not associated with the phenotype Y**.

**Allele frequency**	**Power when the following population stratification is present**			
	**5 and 10%**	**5 and 45%**	**20 and 25%**	**40 and 45%**
Scenario 1: Model 1	0.025	0.070	0.111	0.171
Scenario 1: Model 2	0.064	0.199	0.248	0.394
Scenario 2: Model 1	0.016	0.070	0.103	0.163
Scenario 2: Model 2	0.040	0.205	0.227	0.366
Scenario 3: Model 1	0.025	0.070	0.113	0.172
Scenario 3: Model 2	0.064	0.202	0.249	0.396
Scenario 4: Model 1	0.016	0.064	0.103	0.163
Scenario 4: Model 2	0.040	0.186	0.227	0.366

*Model 1 is used to estimate γ_1_ with a Sidak correction and Model 2 to is used estimate γ_1_ with a screening step where the three SNPs with the largest test statistic given by Equation (4) are tested. Population stratification is present such that half of the subjects have one of the allele frequencies listed and the other half of the subjects have the other allele frequency listed*.

**Table 9:** **This table displays the significance level when one SNP has an indirect effect on the phenotype Y as seen in Figure 2 without the arrow from X to Y and 99 SNPs are not associated with the phenotype Y**.

**Allele frequency**	**Type-1 error rate when the following population stratification is present**			
	**5 and 10%**	**5 and 45%**	**20 and 25%**	**40 and 45%**
Scenario 1: Model 1	0.0011	0.0005	0.0007	0.0003
Scenario 1: Model 2	0.0009	0.0006	0.0008	0.0003
Scenario 2: Model 1	0.0004	0.0015	0.0009	0.0005
Scenario 2: Model 2	0.0003	0.0011	0.0012	0.0005
Scenario 3: Model 1	0.0004	0.0010	0.0008	0.0004
Scenario 3: Model 2	0.0006	0.0009	0.0010	0.0006
Scenario 4: Model 1	0.0008	0.0013	0.0007	0.0004
Scenario 4: Model 2	0.0010	0.0008	0.0011	0.0006

*Model 1 is used to estimate γ_1_ with a Sidak correction and Model 2 to is used estimate γ_1_ with a screening step where the five SNPs with the largest test statistic given by Equation (4) are tested. Population stratification is present such that half of the subjects have one of the allele frequencies listed and the other half of the subjects have the other allele frequency listed*.

**Table 10:** **This table displays the power when one SNP has a direct effect on the phenotype Y and 99 SNPs are not associated with the phenotype Y**.

**Allele frequency**	**Power when the following population stratification is present**			
	**5 and 10%**	**5 and 45%**	**20 and 25%**	**40 and 45%**
Scenario 1: Model 1	0.022	0.050	0.073	0.157
Scenario 1: Model 2	0.044	0.141	0.170	0.324
Scenario 2: Model 1	0.014	0.046	0.071	0.148
Scenario 2: Model 2	0.036	0.137	0.161	0.298
Scenario 3: Model 1	0.022	0.050	0.076	0.159
Scenario 3: Model 2	0.045	0.143	0.174	0.326
Scenario 4: Model 1	0.014	0.046	0.071	0.148
Scenario 4: Model 2	0.036	0.137	0.161	0.298

*Model 1 is used to estimate γ_1_ with a Sidak correction and Model 2 to is used estimate γ_1_ with a screening step where the five SNPs with the largest test statistic given by Equation (4) are tested. Population stratification is present such that half of the subjects have one of the allele frequencies listed and the other half of the subjects have the other allele frequency listed*.

## Data analysis: an application to the framingham study

We evaluated the practical relevance of the proposed adjustment principle by an application to the Framingham Heart Study with 1400 probands (Herbert et al., 2006). For the target phenotype, we selected the lung-function measurement FEV1. For the secondary phenotype K, we selected height. Gender, and age represent *L*, the collection of common risk factors between FEV1 and height. For rs2415815 a SNP associated with both height and FEV1, the test statistic equals 0.044 with corresponding *p*-value equal 0.83. As a result, we fail to reject the null hypothesis and conclude that there is no evidence that the SNP acts directly on FEV1 other than via body height.

## Discussion

Our proposed FBAT assesses the direct genetic effect of a marker locus on the phenotype of interest, other than through another correlated phenotype. The adjustment is based on the conditional mean model approach and can be incorporated into the FBAT-approach in a straightforward fashion. The power of the approach is assessed by simulation studies and shown to be similar to the Vansteelandt et al. method when only one SNP is being tested and superior when multiple SNPs are being tested (Vansteelandt et al., 2009). Unlike the Vansteelandt et al. method, this method uses a screening step and has the unique advantage in situations in which a large number of SNPs are tested for a direct effect on the phenotype of interest. Since the number of tests will be much smaller than the total number of SNPs, this will lead to substantial reduction in the adjustment for multiple-comparisons and will result in improved overall statistical power. In this process, the screening step works under the assumption of no population admixture, but the final analysis of the selected SNPs is robust against it.

While we considered several causal scenarios, if the causal relationships assumed in the DAGs are not true this could cause problems for the proposed method. For example, a causal arrow *K* ← *Y* or *L* → *Y* could introduce spurious association for this method. Therefore, one needs to makes sure that the assumptions of the DAG are met before using the proposed approach. While the simulations considered 50 and 100 SNPs, a realistic application could involve thousands of GWAS SNPs. This leads to extreme multiple test corrections and may lead to very different behavior than the behavior observed in the simulation studies (Morris and Elston, 2011). Furthermore, if phenotypes of the founders are known, the proposed method could perform poorly compared to population-based approaches.

For the screening step in the Simulations section, we chose 3 out of 50 and 5 out of 100 SNPs since this is roughly 5% of the tested SNPs. Another number of SNPs could be chosen for the screening step. Although, if the majority of SNPs are chosen in the screening step (i.e., 40 out of 50 SNPs), this increases the number of multiple comparisons and can decrease power. If too few SNPs are chosen in the screening step (i.e., 1 out of 50 SNPs), this decreases the number of multiple comparisons, but one may fail to detect the causal SNP since too few SNPs were chosen. Care needs to be given to the number of SNPs chosen in the screening step (Van Steen et al., 2005). One cannot simply choose different numbers of SNPs for the screening step until significant results are found since this will inflate the type-1 error rate (Van Steen et al., 2005).

### Conflict of interest statement

The authors declare that the research was conducted in the absence of any commercial or financial relationships that could be construed as a potential conflict of interest.

## Appendix

The following proof shows that the test statistics in the first and second screening steps are uncorrelated under the null hypothesis. As discussed in the introduction and methods sections, $\tilde{Y}=Y-\bar{y}-\gamma_{1}K-\bar{k}$ is the adjusted phenotype for the effect that the phenotype *K* has on the target phenotype *Y*. For ease of notation, we will use $\tilde{Y}=Y-\gamma_{1}(K)$ for this proof. Suppose that the null hypothesis is true that *X* has no effect on *Y* other than through *K*. Let

(9) $$E(Y|X,K,U)=E(Y|K,U)=\Phi \{w(U)+\gamma_{1}K\}$$

where Φ equals the identity link or exponential link and *w*(*U*) is an arbitrary function. Without loss of generality, for the following proof, let Φ equal the identity link. This model does not involve X because we are working under the null hypothesis of no direct effect. Furthermore, the parameter γ_1_ in this model is the same as in model

(10) $$E(Y|X,K,L,S)=w^{*}(X,L,S)+\gamma_{1}K$$

for some function *w*^*^(*X*, *L*, *S*) of (*X*, *L*, *S*), which can be seen by inferring this model from model (9) upon noting that *Y* ⫫ (*L*, *S*)|*X*, *K*, *U* and *U* ⫫ *K*|*L*, *X*, *S*. Using model (9) and model (10) and noting that *Y* ⫫ *S*|*X*, *K*, *U* and *X* ⫫ *U*|*S*, then

(11) $$\begin{array}{l} E[\tilde{Y}(X-E[X|S])]=E[(Y-\gamma_{1}K)(X-E[X|S])]\\ \;=E[w(U)(E[X|S,U]-E[X|S])]=0 \end{array}$$

As a result of Equation (11) and model (9), the $\text{Cov}(\tilde{Y}(X-E[X|S]),\;\tilde{Y}E[X|S])$ can be written as follows

$$\begin{array}{l} \text{Cov}(\tilde{Y}(X-E[X|S]),\tilde{Y}E[X|S])\\ \;=E[{\left(Y-\gamma_{1}K\right)}^{2}E(X|S)(X-E[X|S])]\\ \;=E[{\left(Y-E(Y|X,K,U)+w(U)\right)}^{2}E(X|S)(X-E[X|S])]\\ \;={\text{Part}}_{1}+{\text{Part}}_{2}+{\text{Part}}_{3} \end{array}$$

where

(12) $$\begin{array}{l} {\text{Part}}_{1}=E[\left(w{\left(U\right)}^{2}\right)E[X|S](X-E[X|S])]\\ {\text{Part}}_{2}=E[\left(2(w(U)(Y-E[Y|X,K,U])\right)E[X|S](X-E[X|S])]\\ {\text{Part}}_{3}=E[\left({\left(Y-E[Y|X,K,U]\right)}^{2}\right)E[X|S](X-E[X|S])] \end{array}$$

We will show that the $\text{Cov}(\tilde{Y}(X-E[X|S]),\;\tilde{Y}E[X|S])=0$ by showing that each part of the above equation equals zero.

(13) $$\begin{array}{l} {\text{Part}}_{1}=E[w{\left(U\right)}^{2}E[X|S](X-E[X|S])]\\ \;=E[w{\left(U\right)}^{2}E[X|S](E[X|S,U]-E[X|S])]=0 \end{array}$$

because *X* ⫫ *U*|*S*.

(14) $${\text{Part}}_{2}=E[2w(U)(Y-E[Y|X,K,U])E[X|S](X-E[X|S])]=0$$

because *Y* ⫫ *S*|*X*, *K*, *U*.

(15) $$\begin{array}{l} {\text{Part}}_{3}=E[{\left(Y-E[Y|X,K,U]\right)}^{2}E[X|S](X-E[X|S])]\\ \;=E[E[X|S](X-E[X|S])Var[Y|K,U]] \end{array}$$

because *Y* ⫫ *S*|*X*, *K*, *U* and *Y* ⫫ *X*|*K*, *U*.

Assuming that Var(*Y*|*K*, *U*) is constant, as we do throughout, it is immediate that the term Part_3_ is zero. As a result, this shows that $\text{Cov}(\tilde{Y}(X-E[X|S]),\;\tilde{Y}E[X|S])=0$.
